# Supplementary material for: TOMM40 Genetic Variants Cause Neuroinflammation in Alzheimer’s Disease
Source: Int J Mol Sci. 2023 Feb 17;24(4):4085. doi: 10.3390/ijms24044085 (PMC9962462; doi:10.3390/ijms24044085)
Supplement: Supplementary file 1 [file ijms-24-04085-s001.zip › ijms-2102164-supplementary.pdf]

Supplementary Table S1. List of primary and secondary antibodies used in experiments.

Antibody	Antigen	Host species	Type	Catalog Number	Source
Primary antibody	Iba-1	Rabbit	Polyclonal	10904-1-AP	Proteintech
	COXIV	Rabbit	Monoclonal	#4850	Cell signaling Technology
	β-actin	Mouse	Monoclonal	MAB1501	Merck
	IKK-β	Rabbit	Monoclonal	#8943	Cell signaling Technology
	Phospho-IKKα/β (Ser176/180)	Rabbit	Monoclonal	#2697	Cell signaling Technology
	FLAG	Mouse	Monoclonal	F3165	Sigma
	NF-κB p65	Rabbit	Monoclonal	#8242	Cell signaling Technology
	Phospho-NF-κB p65 (Ser536)	Rabbit	Monoclonal	#3033	Cell signaling Technology
	NLRP3	Rabbit	Monoclonal	#15101	Cell signaling Technology
	ASC/TMS1	Rabbit	Polyclonal	A1170	ABclonal
	Cleaved Caspase-1	Rabbit	Monoclonal	#89332	Cell signaling Technology
Secondary antibody	Goat anti-rabbit IgG, HRP-linked Antibody			#7074	Cell signaling Technology
	Horse anti-mouse IgG, HRP-linked Antibody			#7076	Cell signaling Technology

(A)

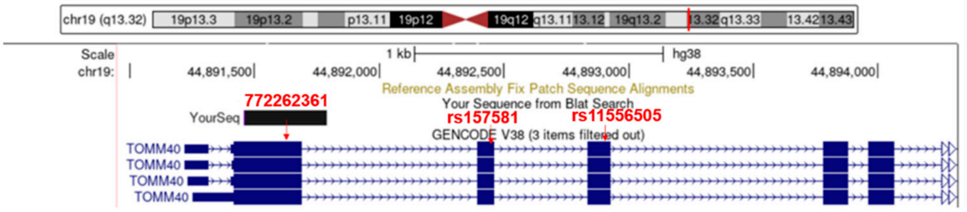

(B)

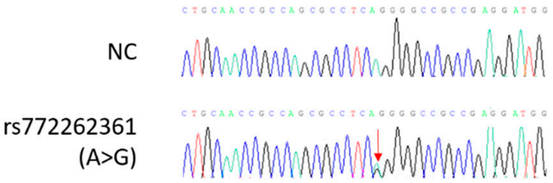

Supplementary Figure S1. TOMM40 SNPs in genomic DNA of AD patients. (A) Physical loci of the associated SNPs within the TOMM40-APOE region. (B) Sanger sequencing confirmed the sequence of rs772262361 from AD patient.
